# Circular RNA hsa_circ_0000034 promotes the progression of retinoblastoma via sponging microRNA-361-3p

**DOI:** 10.1080/21655979.2020.1814670

**Published:** 2020-09-06

**Authors:** Zhe Sun, Ai Zhang, Mingyu Hou, Tao Jiang

**Affiliations:** a Department of Ophthalmology, The Affiliated Hospital of Qingdao University, Qingdao University, Qingdao, Shandong, P.R. China; b Fetal Medicine Center, Qingdao Women and Children’s Hospital, Qingdao University, Qingdao, Shandong, P.R. China; c Department of Anesthesiology, Shanghai East Hospital, Ji’an Hospital, Jiangxi 343000, P. R. China; d Department of Anesthesiology, Shanghai East Hospital, Tongji University School of Medicine, Shanghai 200120, P.R. China

**Keywords:** Retinoblastoma, hsa_circ_0000034, miR-361-3p, progression

## Abstract

Retinoblastoma is the commonest eye cancer occurring in the pediatric population. Circular RNAs (circRNAs) are essential regulators of tumorigenesis and development. The current experiment delves into the function and molecular basis of hsa_circ_0000034 in retinoblastoma progression. In the study, these series of experiments noted an upregulation of hsa_circ_0000034 in retinoblastoma cell lines and tissues. Retinoblastoma patients with raised hsa_circ_0000034 expressions were more likely to possess a more progressive International Integrated Reporting Council (IIRC) stage and optic nerve invasion. hsa_circ_0000034 knockdown caused a marked suppression in the proliferation and invasion of retinoblastoma cells in vitro. Mechanistically, hsa_circ_0000034 appeared to serve as a competitive endogenous RNA (ceRNA) in retinoblastoma through miR-361-3p sponging. In conclusion, our data proved that hsa_circ_0000034 promoted the oncogenicity of retinoblastoma via regulation of miR-361-3p expression, a finding that may contribute toward retinoblastoma therapeutics.

## Graphical abstract

**Figure:**
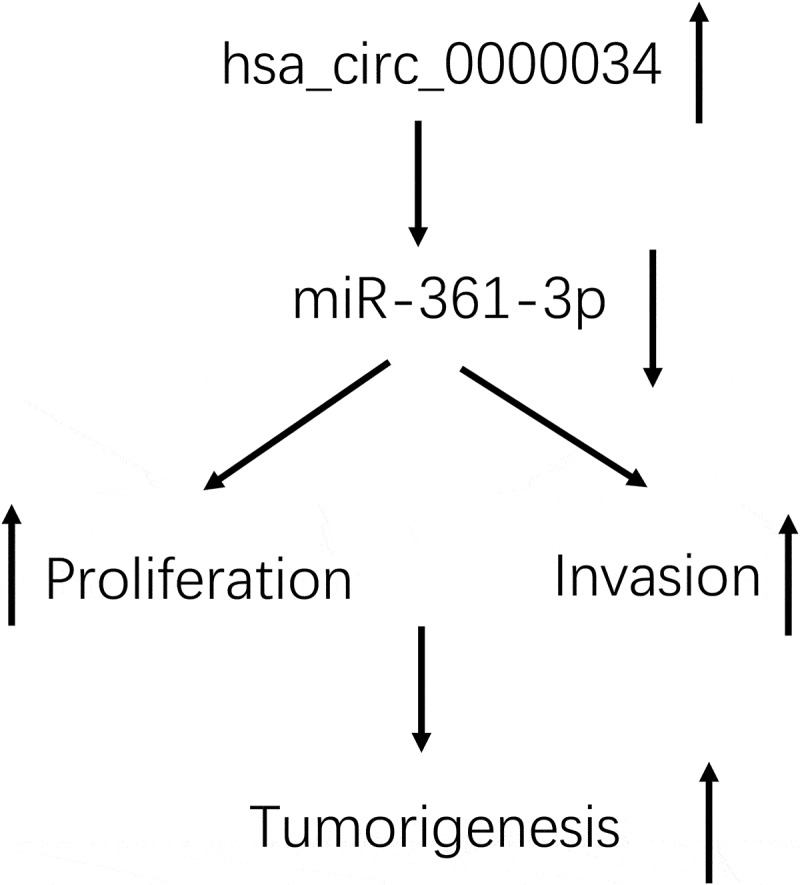

## Introduction

Retinoblastoma is a common malignant eye cancer with the highest incidence in infants and children [1]. With the improvement of combination treatment, the 5-year survival rate of retinoblastoma patients approaches 95% [2]. Nevertheless, the survival rates are still poor in undeveloped countries or regions (less than 30%) [3,4]. It is essential that the full molecular pathway behind retinoblastoma is explored, as this may lead toward breakthroughs in effective therapeutic methods.

Circular RNAs (circRNAs) represent endogenous non-coding RNA possessing a covalent closed-loop structure [5,6]. Increasing papers highlight the participation of circRNAs in several pathological and physiological processes that occur through direct or indirect control of protein expression [7,8]. The aberrant expression of circRNAs is prevalent in a number of human cancer types, including retinoblastoma. For example, Xing et al. [9] demonstrated suppressed retinoblastoma cell growth in the presence of hsa_circ_0001649, as well as induced cell apoptosis through regulation of the AKT/mTOR pathway. Zhao et al. [10] found that circ-0075804 promoted retinoblastoma cell proliferation by forming compounds with HNRNPK to improve the stability of E2F3. Moreover, Chen et al. [11] illustrated that has_circ_0000527 promoted retinoblastoma cell proliferation, invasion and migration through targeting the miR-646/BCL-2 axis. However, the mechanistic and functional characterization of hsa_circ_0000034 in retinoblastoma remain unclear.

Recently, a large number of miRNA molecules play critical roles in the occurrence and progression of retinoblastoma [12]. Li et al. [13] demonstrated that retinoblastoma cell metastasis and proliferation were suppressed by miR-433 mediated depression of Notch1 and PAX6 levels. Bai et al. [14] discovered the pro-growth and anti-apoptotic properties that miR-125b exerted on retinoblastoma cells, which was attributed to suppressed DRAM2 expression. In addition, Zhao et al. [15] unveiled that the expression of miR-361-3p was down-regulated and reduced retinoblastoma cell proliferation and stemness in vitro. However, the mechanistic relationship between miR-361-3p and hsa_circ_0000034 in retinoblastoma has yet to be verified.

The aim of the current study is to explore the roles of hsa_circ_0000034 on the progression of retinoblastoma and reveal the possible mechanisms. Our results demonstrate that upregulated hsa_circ_0000034 was associated with advanced clinical features in retinoblastoma patients and promoted the proliferation and invasion abilities. Furthermore, hsa_circ_0000034 directly sponged miR-361-3p and consequently promoted the progression of retinoblastoma cells in vitro. Thus, hsa_circ_0000034 may act as a potential candidate target in retinoblastoma therapy.

## Materials and methods

### Patients and tissue samples

Thirty-eight retinoblastoma tissue specimens, as well as 12 normal retina tissue specimens, were obtained from the Affiliated Hospital of Qingdao University. All retinoblastoma patients did not receive any therapy before surgery. All experiments were approved by the ethics committee of the Affiliated Hospital of Qingdao University and the signed informed consent had been obtained from all the patients. The clinical features of retinoblastoma patients are shown in Table 1.

**Table 1.** Clinical features of retinoblastoma patients (n = 38).

Clinical features		Case No.
Gender	Male	21
	Female	17
Age	< 2	24
	≥2	14
Tumor size (mm)	< 10	16
	≥10	22
IIRC stage	A-B	11
	C	14
	D-E	13
Optic nerve invasion	Negative	19
	Positive	19

### Cell culture and transfection

The American Type Culture Collection (ATCC, MD, USA) provided the human normal human retinal pigment epithelial cell line (ARPE‐19) and retinoblastoma cell lines (Y79, SO-Rb50 and WERI-Rb1). All specimens were maintained under 5% CO_2_ at 37°C and supplemented with 10% fetal bovine serum (FBS, HyClone, Logan, UT, USA) in RPMI 1640 medium (Invitrogen, Carlsbad, CA, USA).

Small interfering RNA specific to hsa_circ_0000034 (si-circRNA), miR-361-3p mimics, miR-361-3p inhibitors and negative controls were synthesized by GenePharma (Shanghai, China). Transfection was carried out via the lipofectamine 2000 in accordance with the manufacturer’s instruction.

### RNA extraction and real-time PCR (qRT-PCR)

Trizol reagent allowed for extract tissue and cell total RNA which was then used to produce cDNA through reverse transcription with the PrimeScript ^RT^ Master Mix (Takara, Japan). An ABI 7500 Fast Real-Time PCR system (Applied Biosystems) using an SYBR Green PCR Kit (Takara) was used to carry out qRT-PCR. GAPDH, as well as U6, were regarded as controls. The 2^–ΔΔCT^ method allowed for the calculation of the relative gene expression. The primers are as follows: hsa_circ_0000034, 5ʹ-TCCCGTCATGAGATCAGCAAT-3ʹ (forward) and 5ʹ-GCCTGTACAGCTTGTGCAAT-3ʹ (reverse); miR-361-3p, 5ʹ-UCCCCCAGGUGUGAUUCUGAUUU-3ʹ (forward) and 5ʹ- GCAAATCAGAATCACACCTG-3ʹ (reverse) [16].

### Subcellular fractionation location

Isolation of RNA from retinoblastoma cells cytoplasmic and nuclear fractions was done with the Cytoplasmic & Nuclear RNA Purification Kit (Norgen, Belmont, CA, USA). qRT–PCR allowed for quantification of hsa_circ_0000034, GAPDH and U6 in the RNA samples. Internal cytoplasmic reference was GAPDH while U6 represented the nuclear RNA control.

### EdU assay

Retinoblastoma cell proliferation was analyzed using 5-ethynyl-2ʹ-deoxyuridine (EdU) assay Kit (RiboBio) and the previous study [9].

### Wound healing assay

To study retinoblastoma cell migration, wound healing assays were used. Six-well plates were used to house retinoblastoma cells that were plated at 1 × 10^5^ cells/well. Cells were allowed to achieve 85% confluence before being scratched by a sterilized pipette tip. Images were captured by an inverted microscope at each indicated time (0 h and 24 h).

### Transwell invasion assay

Invasion assay was performed via Matrigel-coated membranes. In brief, 1 × 10^5^ retinoblastoma cells were seeded in the top chamber, with the bottom chamber flushed with cell media. Cells were left for 48 h to incubate before the clearance of the cells on the top layer. Cells present in the lower chamber were methanol-fixed before undergoing 0.1% crystal violet staining. Cell numbers were counted using a microscope (Nikon, Japan).

### RNA immunoprecipitation (RIP) assay

The Magna RIP Kit (Millipore, Billerica, MA, USA) was used to perform the RIP assay based on protocols set by the manufacturer. Antibodies against IgG and argonaute 2 (anti-AGO2) were utilized for the RIP assays. Purified RNAs were extracted and the enrichment of hsa_circ_0000034 and miR-361-3p was processed by RT-qPCR.

### Luciferase reporter assay

hsa_circ_0000034-WT (wild-type) or hsa_circ_0000034-MUT (mutant) were inserted into pmirGLO (Promega) to construct 2 reporter plasmids. When retinoblastoma cells reached approximately 70% confluence, Lipofectamine® 2000 was used to transfect the reporter plasmids with either miR-361-3p mimic or inhibitor. The activity of luciferase was determined after 48 h of transfection in compliance with instructions set by the manufacturer.

### Statistical analysis

The SPSS 21.0 (IBM) statistical software was used for this experiment. All data were depicted in terms of mean ± standard deviation (SD) of three separate experiments. Either the Student’s t-test (for two groups) or one-way analysis of variance (ANOVA; for more than two groups) was used to determine intergroup differences. Statistical significance was determined with a p value of <0.05 indicated that the difference was statistically significant.

## Results

### hsa_circ_0000034 expression was upregulated in retinoblastoma

**Figure 1.**
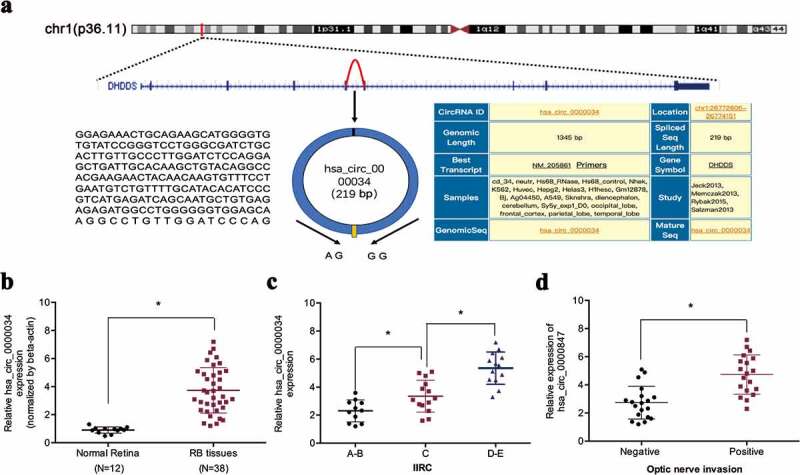
hsa_circ_0000034 expression is upregulated in retinoblastoma. (a) Schematic illustration of hsa_circ_0000034 formation. Arrow indicates back-splicing. (b) hsa_circ_0000034 expression in retinoblastoma tissues. (c, d) High hsa_circ_0000034 expression correlated with advanced IIRC stage and optic nerve invasion in retinoblastoma patients. RB: retinoblastoma. *p < 0.05.

In a previous study, Lyu et al. [15] demonstrated an upregulation of hsa_circ_0000034 in retinoblastoma. However, the mechanistic and functional characterization of hsa_circ_0000034 remain unclear. In our study, we found that hsa_circ_0000034 expressions in retinoblastoma tissues were high in contrast to normal retinal tissues, as determined by qRT-PCR (Figure 1(a,b)). High hsa_circ_0000034 expression in retinoblastoma patients tallied with more progressive IIRC stage and optic nerve invasion (Figure 1(c,d)). Interpreted as a whole, these findings point toward an oncogenic role of hsa_circ_0000034 in retinoblastoma progression.

### Depletion of hsa_circ_0000034 reduced retinoblastoma cells progression

**Figure 2.**
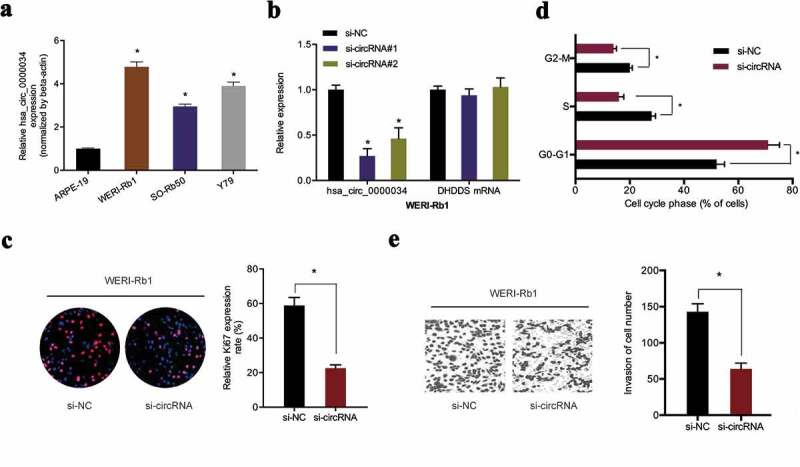
Knockdown of hsa_circ_0000034 inhibited retinoblastoma cells proliferation and invasion. (a) hsa_circ_0000034 expression in retinoblastoma cell lines. (b) qRT-PCR for hsa_circ_0000034 expression by si-circRNA in retinoblastoma cells. (c, d) The proliferation rate and fraction of cell cycle of WERI-Rb1 cells were evaluated using the EdU and flow cytometric assays. (e) The invasion of WERI-Rb1 cells were evaluated by transwell assay. *p < 0.05.

hsa_circ_0000034 was found to be higher in retinoblastoma cell lines in contrast to human normal human retinal pigment epithelial cell line (ARPE‐19) (Figure 2(a)). Next, si-circRNA was transfected into WERI-Rb1 cells with qRT-PCR used to determine the knockdown efficiency (Figure 2(b)). EdU assay revealed that WERI-Rb1 cell proliferation abilities were significantly reduced by si-circRNA transfection (Figure 2(c)). Flow cytometric analysis demonstrated that the loss of hsa_circ_0000034 arrested WERI-Rb1 cells in the G0/G1 phase (Figure 2(d)). In addition, we showed that knockdown of hsa_circ_0000034 decreased the invasion capacities of WERI-Rb1 cells in vitro (Figure 2(e)). These data confirmed the tumor promoter roles of hsa_circ_0000034 in retinoblastoma progression.

### hsa_circ_0000034 served as a sponge of miR-361-3p

**Figure 3.**
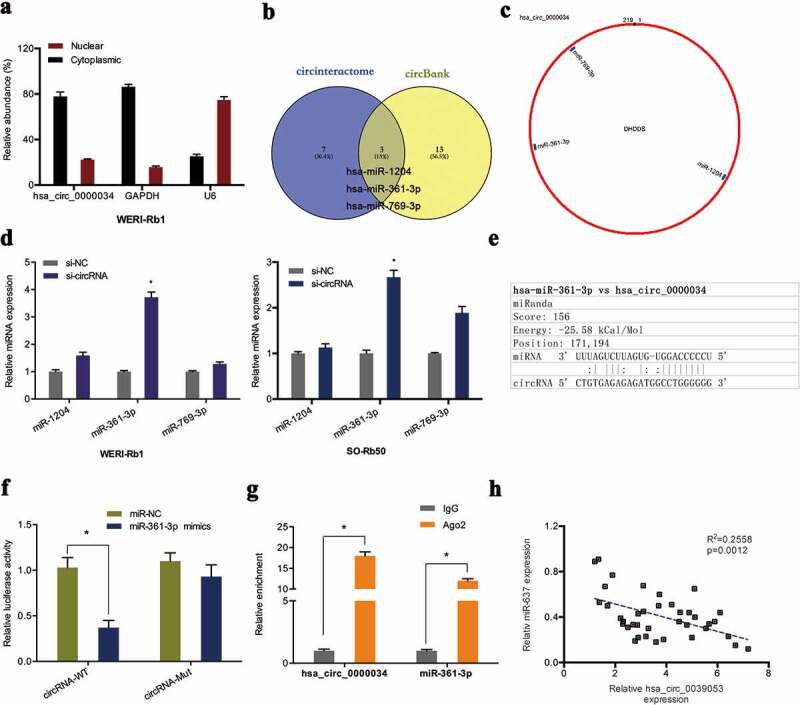
Hsa_circ_0000034 served as a sponge of miR-361-3p. (a) The location of hsa_circ_0000034 in retinoblastoma cells were explored by subcellular fractionation assay. (b, c) Venn diagram showed 3 miRNAs bound to hsa_circ_0000034 in circBank and circinteractome databases. (d) The expression of 3 miRNAs (miR-1204, miR-361-3p and miR-769-3p) in si-circRNA transfected retinoblastoma cells. (e) The putative miR-361-3p binding sites in hsa_circ_0000034. (f, g) Dual-luciferase and RIP assays were used to verify the targeting relationship between hsa_circ_0000034 and miR-361-3p. (h) Upregulated hsa_circ_0000034 levels negatively correlated with miR-361-3p expression in retinoblastoma tissues. *p < 0.05.

In order to delineate how hsa_circ_0000034 contributes to retinoblastoma progression, the molecule was first localized in retinoblastoma cells. Subcellular fractionation assay showed that the molecule was found in highest concentrations in WERI-Rb1 cell cytoplasm (Figure 3(a)). Next, we predicted the miRNA targets by the circBank and circinteractome database. Results showed that miR-1204, miR-361-3p and miR-769-3p might serve as the potential interacting miRNA of hsa_circ_0000034 (Figure 3(b,c)). qRT-PCR showed knocked-down hsa_circ_0000034 significantly raised miR-361-3p expression in retinoblastoma cells (Figure 3(d)). Dual-luciferase reporter assay further demonstrated that miR-361-3p overexpression significantly suppressed the luciferase activity of the hsa_circ_0000034-WT group (Figure 3(e,f)). RIP assay uncovered that hsa_circ_0000034 and miR-361-3p were found in high levels in Ago2-containing beads when contrasted to the IgG group (Figure 3(g)). Furthermore, correlation analysis demonstrated an inverse association between hsa_circ_0000034 expression and miR-361-3p expression in retinoblastoma tissues (Figure 3(h)).

**Figure 4.**
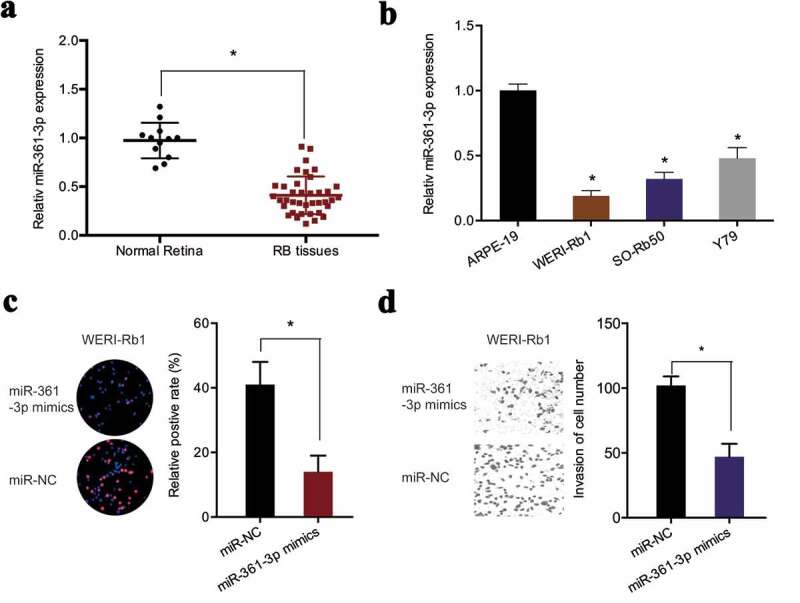
miR-361-3p suppressed retinoblastoma cells progression. (a, b) Expression of miR-361-3p in retinoblastoma tissues and cell lines. (c, d) The proliferation and invasion of retinoblastoma cells were evaluated by EdU and transwell assays. RB: retinoblastoma. *p < 0.05.

Next, miR-361-3p levels were determined in retinoblastoma. qRT-PCR found that miR-361-3p expression was markedly reduced in retinoblastoma tissues and cell lines (Figure 4(a,b)). MiR-361-3p mimics significantly suppressed the proliferation of WERI-Rb1 cells, as demonstrated by EdU assay (Figure 4(c)). Similarly, transwell assay revealed that miR-361-3p upregulation obviously inhibited the invasive abilities of WERI-Rb1 cells (Figure 4(d)). Collectively, the findings suggested that hsa_circ_0000034 might function as a sponge of miR-361-3p in retinoblastoma cells.

### miR-361-3p downregulation reversed the effects of hsa_circ_0000034 knockdown in retinoblastoma

**Figure 5.**
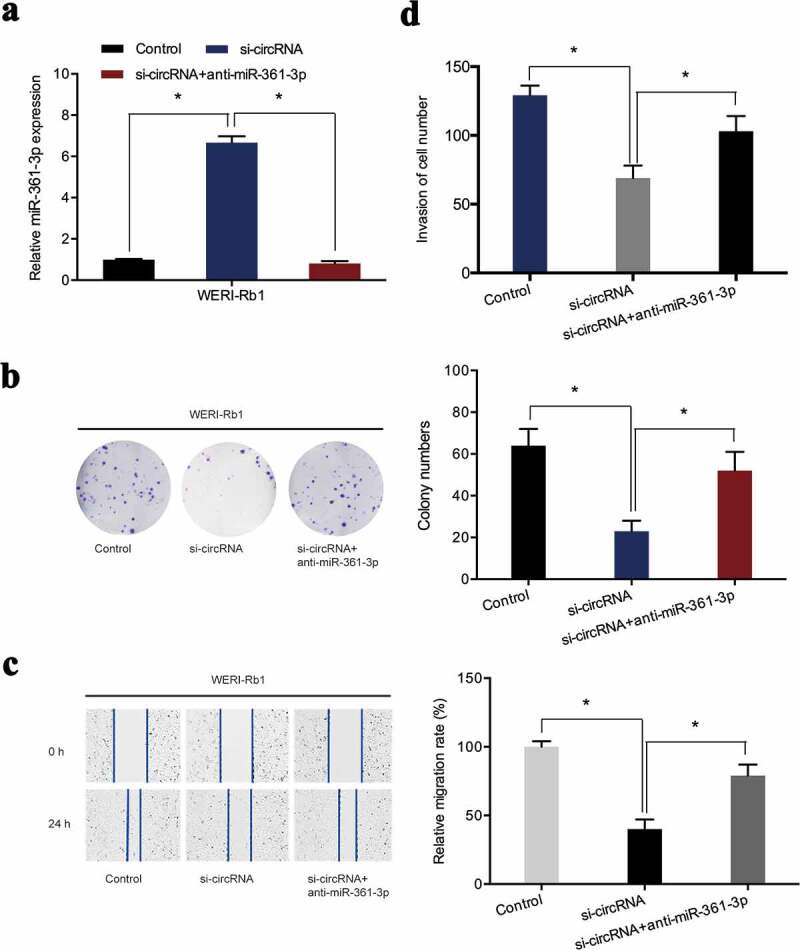
miR-361-3p inhibitors abrogated the regulatory effects of si-circRNA on WERI-Rb1 cells. (a) miR-361-3p expression was assessed in retinoblastoma cells post-introduction of miR-361-3p inhibitors and si-circRNA. (b) Colony formation assay revealed that miR-361-3p inhibitors abolished the roles of si-circRNA on WERI-Rb1 cells proliferation. (c, d) Wound healing and transwell assays showed si-circRNA-induced suppression of the migratory and invasive properties of WERI-Rb1 cells were reversed by miR-361-3p inhibitors. *p < 0.05.

To further confirm that hsa_circ_0000034 exhibited oncogenic effects on retinoblastoma progression through sponging miR-361-3p, miR-361-3p inhibitors were co-transfected with si-circRNA into retinoblastoma cells (Figure 5(a)). Colony formation assay revealed that miR-361-3p inhibitors rescued the proliferative activity of WERI-Rb1 cells suppressed by hsa_circ_0000034 inhibition (Figure 5(b)). The inhibiting influence of si-circRNA on WERI-Rb1 cells migratory capabilities was reversed after co-transfection with the miR-361-3p inhibitors (Figure 5(c)). Furthermore, si-circRNA-induced suppression of WERI-Rb1 cell invasion was also abolished by miR-361-3p inhibitors (Figure 5(d)). Collectively, we found that hsa_circ_0000034 performed its pro-oncogenic roles in retinoblastoma cells by regulating miR-361-3p expressions.

## Discussion

Increasing evidence has indicated that dysregulated circRNAs contribute to carcinogenesis and tumor progression [17]. Recently, several studies reported that circRNAs might serve as either anti-tumor or tumor-promoting agents in retinoblastoma progression. For example, Zhang et al. [18] showed that circ_0000527 could enhance retinoblastoma cell proliferation and metastasis by regulating the miR-646/LRP6 axis. Xu et al. [19] suggested that CircVAPA regulated the development of retinoblastoma by acting as a sponge of miR-615-3p to control SMARCE1. Lyu et al. [15] showed that has_circ_0093996 acts as a ceRNA to promote retinoblastoma progression via miR-183/PDCD4 axis. Therefore, circRNA plays an important function in the therapy and prognosis of retinoblastoma. In our study, we firstly revealed the function of hsa_circ_0000034 in retinoblastoma. The current investigations demonstrated a positive association between hsa_circ_0000034 overexpression to more progressive IIRC stage and optic nerve invasion in retinoblastoma patients. Functionally, the inhibition of hsa_circ_0000034 reduced retinoblastoma cell viability and invasive abilities in vitro. The findings indicate that hsa_circ_0000034 could accelerate the progression of retinoblastoma.

Recently, numerous studies have revealed that circRNAs indirectly regulate gene expression by serving as competing endogenous RNAs (ceRNAs) [20,21]. Li et al. [22] showed that circRBMS3 downregulation inhibited gastric cancer cell invasion and proliferation through sponging miR-153 and regulating SNAI1 expression. Chen et al. [23] found that circular RNA 100,146 functioned as an oncogene by directly binding to miR-361-3p and miR-615-5p in lung cancer. Liu et al. [24] suggested that circSERPINA3 promoted cell proliferation and invasion in nasopharyngeal carcinoma by regulating miR-944/MDM2 axis. Accordingly, we suspected that hsa_circ_0000034 works through such a ceRNA to sponge miRNAs.

To verify our hypothesis, bioinformatics analyses were conducted to search for miRNAs that may bind to hsa_circ_0000034, and miR-361-3p was found to be a potential candidate. qRT–PCR analysis uncovered that hsa_circ_0000034 knockdown increased miR-361-3p expressions in retinoblastoma cells. RIP and luciferase reporter assay further confirmed the relationship between miR-361-3p and hsa_circ_0000034. In addition, rescue assays demonstrated that suppressing miR-361-3p reversed the effects of hsa_circ_0000034 knockdown on retinoblastoma cell progression. Collectively, these results suggested that hsa_circ_0000034 might promote the malignancy of retinoblastoma cells by sponging miR-361-3p.

## Conclusion

In summary, our findings demonstrated that hsa_circ_0000034 promoted retinoblastoma cell metastasis and growth in vitro by sponging miR-361-3p, which might provide a valuable insight into this molecule as a therapeutic target for retinoblastoma treatment. However, our research has the following limitations, first, the number of patients is not big enough, and we will increase the number of patients in further study. Second, the effects and underlying mechanisms of hsa_circ_0000034 in vivo are still needed to be explored.

## Highlights

1. hsa_circ_0000034 is upregulated in retinoblastoma.
2. hsa_circ_0000034 promotes retinoblastoma cell proliferation and invasion abilities in vitro.
3. hsa_circ_0000034 functioned as a sponge of miR-361-3p in retinoblastoma cells.
